# Carbohydrate and Amino Acid Metabolism as Hallmarks for Innate Immune Cell Activation and Function

**DOI:** 10.3390/cells9030562

**Published:** 2020-02-27

**Authors:** Haoxin Zhao, Lydia N. Raines, Stanley Ching-Cheng Huang

**Affiliations:** 1 Department of Pathology, Case Western Reserve University School of Medicine, Cleveland, OH 44106, USA; hxz619@case.edu (H.Z.); lxr220@case.edu (L.N.R.); 2 Case Comprehensive Cancer Center, Case Western Reserve University School of Medicine, Cleveland, OH 44106, USA

**Keywords:** innate immunity, carbohydrates, amino acids, immunometabolism

## Abstract

Immune activation is now understood to be fundamentally linked to intrinsic and/or extrinsic metabolic processes which are essential for immune cells to survive, proliferate, and perform their effector functions. Moreover, disruption or dysregulation of these pathways can result in detrimental outcomes and underly a number of pathologies in both communicable and non-communicable diseases. In this review, we discuss how the metabolism of carbohydrates and amino acids in particular can modulate innate immunity and how perturbations in these pathways can result in failure of these immune cells to properly function or induce unfavorable phenotypes.

## 1. Carbohydrate and Amino Acid Metabolism

The field of immunometabolism has grown significantly over the past several decades, perhaps driven by the realization that cellular metabolism is fundamental to the activation and effector function of all cells within the body. While early links between immunity and metabolism were uncovered in the late 1900s, it was not until the early 2000s when it was observed that macrophages within the adipose tissue of obese mice exhibited an upregulation of inflammatory gene expression that this association was fully appreciated [1,2]. Since then, detailed reports into the activation and effector function of these adipose tissue-associated macrophages have paved the way to insights into how cellular metabolism affects other immune cell subtypes as well as how these signaling cascades influence global changes in these cells. Here, we discuss how carbohydrate and amino acid metabolism shape phenotypic outcomes in innate immune cells.

### 1.1. Carbohydrate Metabolism

**Figure 1:**
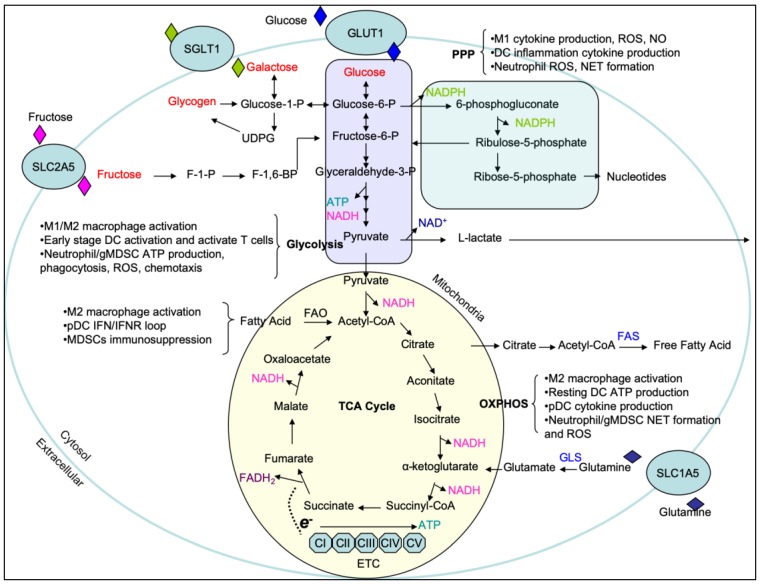
Overview of major carbohydrate metabolic pathways. Carbohydrates are highlighted in red with the major contributions of the respective pathways described in the brackets. ETC, electron transport chain; F-1-P, fructose-1-phosphate; F-1,6-BP, fructose 1,6-bisphosphate; FAD, flavin adenine dinucleotide; FAS, fatty acid synthase; FAO, fatty acid oxidation; GLS, glutaminase; GLUT1, glucose transporter 1; NAD, nicotinamide adenine dinucleotide; OXPHOS, oxidative phosphorylation; PPP, pentose phosphate pathway; SGLT1, sodium/glucose con-transporter 1; SLC1A5, neutral amino acid transporter family 1 member 5; SLC2A5, neutral amino acid transporter family 2 member 5; TCA, tricarboxylic acid cycle or Krebs cycle; UDPG, uridine diphosphate-glucose.

Perhaps the most well-known metabolic pathway is glycolysis. Glycolysis begins with the acquisition of glucose through glucose transporters (e.g., GLUT1) (Figure 1). Upon entering the cell, glucose is catalytically broken down in the cytosol via an ATP-dependent hexokinase reaction into the first product of the glycolytic pathway glucose-6-phosphate (G6P). G6P is further metabolized to the eventual final glycolytic product pyruvate, resulting in a net gain of 2 ATP [3,4]. In parallel to glycolysis, G6P can also enter the pentose phosphate pathway (PPP) in the cytosol, where G6P is converted into ribose for RNA synthesis, NADPH production and reactive oxygen species (ROS) generation. This pathway is also necessary to reduce glutathione (GSH), an antioxidant and ROS scavenger that is crucial to protect pro-inflammatory immune cells from incurring damage from increased ROS production [5]. In the oxidative phase, G6P is converted to ribulose-5-phosphate by 6-phosphogluconate dehydrogenase (PGD) for NADPH production, which is not only used by NADPH oxidase (NOX) to generate ROS [6], but is also utilized for fatty acid biosynthesis in the prostaglandin production, plasma membrane synthesis, and phagocytic function in phagocytic cells [7,8]. Furthermore, the generation of ribose-5-phosphate serves as a precursor for nucleotides and amino acids, which have roles in many other biosynthetic functions that will be described below. Pyruvate can enter the mitochondria and be catabolized by pyruvate dehydrogenase into acetyl-CoA. Acetyl-CoA shuttles into the tricarboxylic acid (TCA) cycle as fuel for the production of citrate, isocitrate, alpha-ketoglutarate (α-KG), succinyl-CoA, succinate, fumarate, malate, and oxaloacetate (Figure 1). While successful completion of the TCA cycle and electron transport chain results in a net gain of 36 molecules of ATP, these TCA cycle intermediates are also known to participate in metabolic processes outside of ATP production. Prominent examples of this are citrate and succinate metabolism. Citrate, produced from oxaloacetate and acetyl-CoA, can escape the mitochondria through the mitochondrial citrate carrier (SLC25A1). Once in the cytosol, citrate can be used in the production of nitric oxide (NO), ROS, prostaglandin E2 (PGE2), and cytosol acetyl-CoA which are crucial for modulating inflammatory responses [9,10]. Similarly, succinate entering the cytosol can be used as a cue to inhibit the activity of HIF-1α prolyl hydroxylase (PHD) and aid HIF-1α-stabilization. This in turn, increases expression of glycolytic machinery during inflammation [11,12]. Thus, TCA cycle is one point of disparity amongst pro-inflammation and anti-inflammation in immune cells which will be discussed in detail below.

TCA cycle generates the reducing equivalents NADH and FADH2 which are essential to support the activity of mitochondrial respiratory chain, also known as the electron transport chain (ETC). The ETC is composed of four large multiprotein complexes (complex I to IV), and two diffusible electron carriers (cytochrome C and ubiquinone) in the inner membrane of mitochondrion. It is known that Toll-like receptor (TLR) activation via tumor necrosis factor receptor-associated factor 6 (TRAF6) translocation to the mitochondria interacts with evolutionarily conserved signaling intermediate in the Toll pathway (ECSIT) to promote mitochondrial ROS (mtROS) production and the recruitment of the mitochondria to phagosomes [13]. The complexes of the ETC except for complex II (or Succinate dehydrogenase (SDH)) are able to form supercomplexes in the mitochondrial inner membrane which limit excessive mtROS formation from the respiratory chain [14]. The oxidation of succinate into fumarate by SDH is coupled to the reduction of ubiquinone (UQ) to ubiquinol (UQH2). However, high levels of succinate oxidation favor the process of reverse electron transport (RET) at complex I, which is associated with significant release of mtROS leading to oxidative damage and cell death [12,15]. Rotenone or the diabetic drug metformin can suppress the activity of ETC complex I and markedly impair the production of mtROS, as well as IL-1β to abate inflammation [16]. Inhibition of complex II (SDH) by dimethyl malonate (DMM) displays a similar effect having reduced IL-1β but increase IL-10 production [16].

Glucose can also polymerize to form larger polysaccharides such as glycogen [17]. Glycogen has been extensively studied in hepatocytes, muscle cells, and neuronal tissue in which it serves as an intracellular cytosolic carbon reservoir [18,19,20,21]. A number of enzymes are required for the synthesis of glycogen in cells. As previous described, glucose taken into the cell can be phosphorylated by hexokinase into G6P and then isomerized by phosphoglucomutase-1 into glucose-1-phosphate. Glucose-1-phosphate is catalyzed by UDP-glucose pyrophosphorylase to become uridine diphosphate-glucose (UDPG) for the construction of glycogen [22,23,24] (Figure 1). Glycogen breakdown occurs in the cytoplasm and also the lysosomes. In the cytosol, glycogen is catabolized by the enzymes glycogen phosphorylase and glycogen debranching enzyme to generate free glucose. Glucose-1-phosphate derived from glycogen in the cytosol may be isomerized into glucose 6-phosphate and dephosphorylated to free glucose by glucose-6-phosphatase (G6Pase) [18,25]. Moreover, lysosomal acid α-1,4-glucosidase, 1,4-α-glucan hydrolase, and/or acid maltase primarily hydrolyze the branched polymer points of glycogen in the lysosomes to release glucose [26]. The regulation of glycogen-glucose homeostasis is mediated by G6Pase complex, including hydrolase and glucose-6-phosphate transporter (G6PT) subunits. It has been reported that G6PT deficiency causes glycogen storage diseases (GSD) and develops myeloid progenitor dysregulation and neutrophilia with tendency to bacterial infections [27]. Patients with G6PT deficiency tend to be at risk for inflammatory bowel disease (IBD) and autoimmune endocrine disorders [28]. Furthermore, deficiency of G6PT impairs glucose homeostasis reducing glucose uptake and cellular levels of G6P, lactate, ATP, and NADH of cells [27].

Galactose is a monosaccharide hydrolyzed from lactose by α-galactosidase, which can be utilized and transported across the cell membrane via the Na^+^/glucose co-transporter or symporter sodium/glucose con-transporter 1 (SGLT1) in cells [29]. Galactose exists in two forms, the α- and β-pyranose structures. Upon its release from lactose, galactose exists in a β-conformation and is then converted to α-galactose by galactose mutarotase prior to entering the Leloir pathway. Galactokinase catalyzes the first step of galactose phosphorylation generating galactose-1-phosphate (Gal-1-P) at the expense of one ATP [30]. Then galactose-1-phosphate uridylyltransferase (GALT) converts Gal-1-P into uridine diphosphate galactose (UDP-gal), with the UDP derived from UDP-glucose (UDPG), and as a result glucose-1-phosphate is generated. UDP-gal can be converted to UDPG for glycosylation reactions and glucose-1-phosphate can be further metabolized to form G-6-P for glycolysis respectively [31] (Figure 1). The conversion of galactose to glucose can modulate the effector function of immune cells [32,33]. It has been reported that deficient GALT activity results in a marked accumulation of galactose and Gal-1-P in tissues leading to inflammatory dysregulation and developmental disorder of galactosemia [34,35].

Fructose appears as uniquely contributed to obesity and obesity-related cardiometabolic complications [36,37], and its metabolism greatly differs from glucose. Fructose is predominantly taken up by the transporter SLC2A5 (GLUT5) [38] (Figure 1). In the tissues of muscles, adipose tissue and kidney, fructose is phosphorylated to fructose-6-phosphate (F-6-P) by hexokinase (HK) in the glycolytic pathway. However, in the liver, fructose is first phosphorylated by glucokinase (GK) to fructose-1-phosphate (F-1-P) and is further converted to produce dihydroxyacetonephosphate (DHAP) and glyceraldehyde (GA). Catabolism of GA is phosphorylated to glyceraldehydes-3-phosphate (GA3P) and joins to DHAP to make fructose-1,6-bisphosphate (F-1,6-BP) into the metabolite pools of glycolysis [39]. In parallel, DHAP can also be converted to glycerol-3-phsophate, which can be packaged into very-low density lipoproteins (VLDL). Therefore, excessive intake of fructose may create an unfavorable lipid profile leading to metabolic disorders [40]. Fructose-induced obesity exhibits higher levels of chronic inflammation and accumulated macrophages in adipose tissues. Importantly, it has been demonstrated that macrophages are responsible for the production of TNF-α, IL-6, NO, and IL-1β in adipose tissues of the obese [1,41], and these secreted pro-inflammatory cytokines seem to be associated with pathogenesis of hepatic insulin resistance and the progression of fatty liver [42]. Furthermore, high fructose intake induces a metabolic shift in human dendritic cells towards glycolysis and cell activation by promoting IL-6 and IL-1β production [43]. Increased numbers of myeloid cells and induction of TLRs and TLR-dependent pathways (e.g., MyD88, iNOS, and TNF-α) were found to present in liver with fructose-induced hepatic steatosis [44]. Collectively, high fructose environment appears to affect metabolic reprogramming and pro-inflammatory activation of immune cells and defining the molecular compartments and mechanisms of which may offer potential therapeutic targets for lessening fructose-induced inflammation.

### 1.2. Amino Acid Metabolism

**Figure 2:**
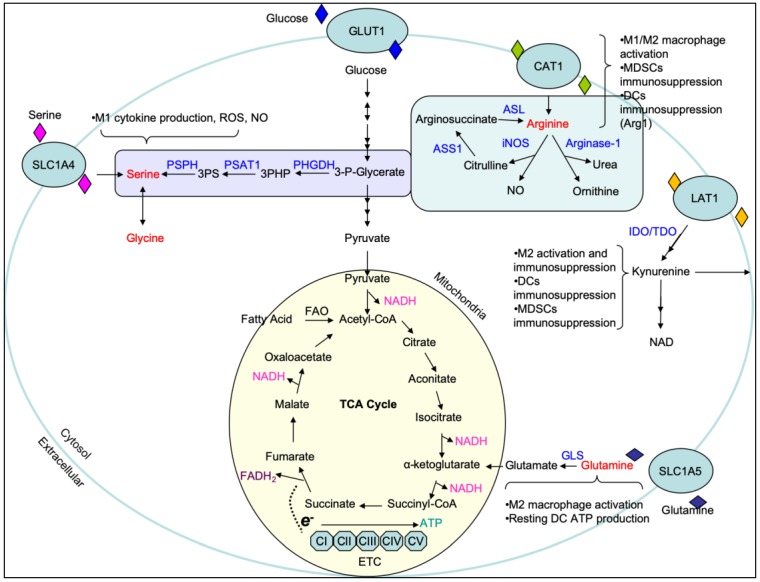
Overview of major amino acid metabolic pathways. Amino acids are highlighted in red with the major contributions of the respective pathways described in the brackets. Key enzymes are highlighted in blue. 3PHP, 3-phosphohydroxypyruvate; 3PS, 3-phosphoserine; ASL, argininosuccinate lyase; ASS1, argininosuccinate synthetase; CAT1, cationic amino acid transporter 1; IDO, Indoleamine-pyrrole 2,3-dioxygenase; iNOS, induced nitric oxide synthase; FAD, flavin adenine dinucleotide; FAO, fatty acid oxidation; GLS, glutaminase; NAD, nicotinamide adenine dinucleotide; NO, nitric oxide; OXPHOS, oxidative phosphorylation; PHGDH, phosphoglycerate dehydrogenase; PSAT1, phosphoserine aminotransferase; PSPH, phosphoserine phosphatase; ROS, reactive oxygen species; TCA, tricarboxylic acid cycle or Krebs cycle; TDO, tryptophan 2,3-dioxygenase.

Amino acids, in addition to carbohydrates, have recently been deemed necessary for the development of immune cells, particularly in polarization and effector function [45,46,47]. Arginine, a non-essential amino acid, is the precursor for ornithine, citrulline, and nitrite in macrophages [48]. Later, NO was identified as the active intermediate of the arginine to nitrite pathway in cells [49,50,51]. Arginine can both be synthesized de novo [52,53] or taken up from the extracellular environment through the arginine transporter CAT-1 [54,55] (Figure 2). *De novo* synthesis of arginine is a means through which excess citrulline can be scavenged to produce more arginine for nitric oxide species (NOS) generation and requires the amino acid aspartate [52,53]. The conversion of citrulline to arginine is an energy intensive process which requires the hydrolysis of ATP to AMP. After acquiring ATP and becoming the reactive intermediate citrulline adenylate, it undergoes a rate-limiting reaction catalyzed by arginosuccinate synthetase (ASS1) that requires aspartate and liberates AMP to produce arginosuccinate. Arginosuccinate is then converted into arginine by arginosuccinate lyase (ASL), freeing fumarate as a byproduct. Once acquired, arginine is further catabolized into ornithine and urea by arginase-1 (Arg1) or into NO and citrulline by iNOS. Both ornithine and citrulline can be shuttled into downstream metabolic pathways such as the uric acid cycle. The upregulation of iNOS and arginase-1 are strongly associated with the activation state of macrophages and are intimately integrated with immune responses under physiological and pathological conditions [56,57,58].

While arginine metabolism serves as a classic example of how amino acids can differentially regulate immune cell function, glutamine is perhaps the most well studied. Glutamine is a non-essential amino acid used predominantly in nucleotide synthesis, energy production, and a majority of the biosynthetic pathways associated with cell proliferation and biological function [59,60,61,62]. Moreover, glutamine metabolism funneling into arginine synthesis is known to be important for the production of NO [63]. Glutamine has emerged as a key regulatory component necessary for numerous aspects of adaptive and innate immune function in the inflammatory microenvironment [46,64]. Inhibition or deficiency of glutamine metabolism results in dysregulation/unbalance of activation and/or development in macrophage, Th17, regulatory T (Treg), and B cells, respectively [63,65,66,67,68,69,70]. The acquisition of glutamine occurs either through uptake from the extracellular space or via de novo synthesis. Glutamine intake can occur through a wide array of amino acid transporters such as SLC1A5 [71] and used for downstream biosynthetic reactions or shuttled back out of the cell via glutamine antiporters for use as an amino acid exchange factor [72]. This exchange capacity allows cells to acquire essential amino acids such as leucine which cannot be synthesized de novo [72]. In contrast to glutamine intake, glutamine synthesis occurs in the mitochondria by an ATP-dependent conversion of ammonia and glutamate driven by the enzyme glutamine synthetase (GS) [73,74]. Further, intracellular glutamine can be hydrolyzed by glutaminase (GLS) to generate glutamate as a critical step to the TCA cycle entry at α-KG [75] (Figure 2).

Recently, important epigenetic roles for serine and glycine have been uncovered in a number of immune cells as well as cancer. In T cells, serine was found to be essential for proliferation by supporting purine biosynthesis [76]. Moreover, impaired serine/glycine metabolism led to compromised naïve T cell activation [77]. The role for serine and glycine in innate immune cells has not been fully elucidated, but what has been uncovered will be discussed below. Immune cells can acquire serine through de novo synthesis or extracellular uptake [78]. Briefly, de novo synthesis of serine is an offshoot of glycolysis in which the glycolytic intermediate 3-phosphoglycerate is enzymatically converted into serine. This process occurs in three steps, with the rate-limiting step being the conversion of 3-phosphoglycerate into 3-phosphohydroxypyruvate via phosphoglycerate dehydrogenase (PHGDH). 3-phosphohydroxypyruvate is then converted into 3-phosphoserine by phosphoserine aminotransferase (PSAT1) and requires the transamination of glutamate into α-KG. The last step converts 3-phosphoserine into serine by phosphoserine phosphatase (PSPH). Notably, the conversion of serine into glycine occurs in the mitochondria and is the beginning step of one-carbon metabolism which provides one-carbon units into the folate cycle and methionine cycle leading to methylation reactions, purine synthesis, and redox homeostasis.

Unlike the other amino acids described in this review, tryptophan is an essential amino acid that can only be acquired through dietary intake. Once acquired by the cell, tryptophan can be shuttled into two major pathways, the serotonin pathway and the kynurenine pathway, where it acts as a precursor for the synthesis of biologically essential compounds [79,80,81]. The role of tryptophan metabolism has been well studied in the context of neurological research due to its necessity in serotonin synthesis; however, it has recently become of interest in the context of tumor biology due to the production of kynurenine, a potent T and NK cell immunosuppressant. Kynurenine is the central metabolite of the kynurenine pathway but is not the final product; this is nicotinamide adenine dinucleotide (NAD). Briefly, tryptophan is catabolized by the enzyme indoleamine 2,3-dioxygenase (IDO) into N-formyl-kynurenine which is then converted into kynurenine. IDO is expressed on a number of immune cells including macrophages and dendritic cells and has been found to modulate T cell behavior. Increased IDO, coupled with increased kynurenine production, has been reported to suppress antitumor responses and aid in tumor progression.

## 2. Regulation of Carbohydrate and Amino Acid Metabolism in Innate Immunity

### 2.1. Macrophages—M1 Macrophages

As resident cells of almost every tissue in the body, macrophages play critical roles in defense against pathogens through phagocytic and inflammatory activities, and maintenance of tissue homeostasis [82]. Macrophages adopt different functional phenotypes with distinct gene expression profiles and functions due to their plasticity. These result in the pro-inflammatory (or classically activated; M1) and anti-inflammatory (or alternatively activated; M2) distinctions [83,84,85]. How macrophages adopt these distinct pathways have been the subject of study for many years, and it is now recognized that cellular metabolism plays an essential role in determining cell fate [57,86,87]. For instance, M1 macrophages, upon stimulation with TLR agonists in combination with interferon-γ (IFN-γ), employ aerobic glycolysis rather than mitochondrial oxidative phosphorylation (OXPHOS) to increase the dependency of glucose for rapid ATP production [86]. Glucose is poorly oxidized, but is converted into lactate [88], by which this process provides key metabolic intermediates (e.g., NADH) for biosynthetic pathways to support M1 macrophage activation [89], and also pro-inflammatory cytokine secretion and phagocytosis [86,90,91,92] (Table 1).

Stimulation of TLR signaling in macrophages is known to induce activation of hypoxia-inducible factor 1α (HIF-1α), a key transcription factor regulating inflammation in a number of different contexts [11], and which acts as a metabolic switch to induce glycolytic gene expression, such as GLUT1 [93], phosphoglyceratekinase (PGK) [94], and lactate dehydrogenase (LDH) [95]. Increased expression of HIF-1α has been shown to induce the expression of iNOS, an important enzyme for arginine metabolism which generates NO to support macrophage phagocytic activity [56]. However, inhibition of glycolysis with 2-DG can hinder HIF-1α activation and impairs cytokine production of IL-1β and TNF-α in M1 macrophages [8,94,96]. HIF-1α-deficient M1 macrophages exhibit decreased iNOS expression [96] and are less capable of curbing bacterial infection [97]. The mechanistic target of rapamycin (mTOR) is known to control the activation of HIF-1α signaling to fine-tune the metabolism of glucose and has been reviewed extensively elsewhere [98,99,100].

In line with increased glucose dependency, another metabolic hallmark of TLR-stimulated macrophages is to shutdown reactions of mitochondrial oxidative metabolism, and induce reverse electron transport (RET) [12,57,101]. Recently, it has been reported that the glycolytic intermediate glycerol 3-phosphate dehydrogenase (GPD2) oxidizes glycerol-3-phosphate (G3P) to dihydroxyacetone phosphate (DHAP) to facilitate the generation of mitochondrial electrons and also to induce glucose oxidation for acetyl-CoA production [102]. Furthermore, the activity of the pentose phosphate pathway (PPP), a metabolic pathway parallel to glycolysis is enhanced in LPS-stimulated macrophages to support nucleotide synthesis and nicotinamide NADPH used for ROS production and fatty acid synthesis [66,103]. Inhibition of 6-phosphogluconate dehydrogenase (PGD) in the PPP was demonstrated to suppress pro-cytokine secretion and also the inflammatory response of macrophages stimulated with LPS [7]. Conversely, carbohydrate kinase-like protein (CARKL) involved in the conversion of sedoheptulose into sedoheptulose-7-phosphate in the PPP is repressed in M1 macrophages, suggesting the expression of CARKL is responsible for reprogramming glucose metabolism and activation states of macrophages during inflammation [103].

Glucose can be obtained from glycogen degradation in cells. It has been indicated that overexpression of glucose transporter (GLUT1) will elevate the metabolic rate of glucose and increase lactate production, glucose oxidation, and also glycogen storage in macrophages [92]. Indeed, glycogen exhibits immunomodulatory activity that was found to interact with TLR2 to upregulate iNOS expression and the production of NO and inflammatory cytokines (e.g., IL-6 and TNF-α) [104,105]. Moreover, glycogen negatively regulates oxidative stress and cytokine secretion from intestinal macrophages, which mitigates mouse colitis [106]. Similarly, oral administration of glycogen has been found to increase natural killer cell activity and promote anti-tumor activity in mice [107].

In addition to changes in glucose metabolism, it is known that the availability of arginine is one of the rate-limiting factors for generating NO, which is a pivotal pro-inflammatory effector for macrophages to control and eliminate intracellular pathogens and/or tumor cells [56]. As a byproduct of arginine metabolism, citrulline can be recycled to arginine via the regulation of ASS1 and ASL of the citrulline-NO cycle to generate NO [108,109]. It has been shown that macrophages require ASS1 to import citrulline to synthesize arginine and sustain NO output as ASS1 deficiency in macrophages fails to control mycobacterial infection in animal models [110]. In contrast, M2 macrophages upregulate Arg1, breaking down arginine into L-ornithine and urea [111,112]. Arg1 activity is required for local tissue wound healing [113], and its expression is greatly increased by Th2 cytokine signaling through STAT6 in M2 macrophages associated with anti-helminth functions [114]. However, Arg1 expression can also be induced by TLR-mediated signaling independent of STAT6 [115]. Inhibition of macrophage Arg1 produces more NO to eliminate mycobacterial infection [115], but prolongs local tissue inflammation and wound repair in animals [113].

In addition to arginine, increased glutamine is crucial for anaplerosis of the TCA cycle in macrophages. The levels of glutamine-derived α-KG were found to contribute to M2 macrophage polarization, which mechanistically suppresses IKK activation required for NF-κB mediated pro-inflammatory effects [65]; however, reduced cellular level of α-KG aids in the accumulation of succinate to stabilize the activity of HIF-1α for the expression of M1-specific genes and enzymes required for glycolysis and effector function [8,94,116].

### 2.2. Macrophages—M2 Macrophage

In contrast to pro-inflammatory M1 macrophages, M2 macrophages are canonically considered to be anti-inflammatory effector cells which function in a range of physiological and pathological processes, including tissue homeostasis, wound healing, helminth infection, and also malignancy (Table 1) [82,117,118,119]. Metabolic reprogramming in activated M2 macrophages is characterized by an intact TCA cycle and enhanced mitochondrial respiration, mitochondrial mass, and fatty acid oxidation (FAO) [89,120,121]. This continuous flux of metabolites through the TCA cycle is crucial to meet the energy demands of M2 macrophages required for cell proliferation and glycosylation of lectin and mannose receptors [66,122,123].

Glucose utilization through aerobic glycolysis is known to be essential for M1 macrophages; however, it has been suggested that glucose metabolism is also important for protumoral (or M2) macrophages [122,124]. While less than M1, M2 macrophages exhibit higher glucose uptake, expression of genes encoding glycolytic enzymes and lactic acid production than naïve macrophages. Therefore, both M1 and M2 macrophages require glycolytic metabolism but at varying degrees to support their immunological activity [122,124].

Metabolic intermediates generated from the TCA cycle can be signal transducers participating in alternative pathways to modulate cell fate and function in macrophages and also other immune cells [125,126]. For instance, the TCA intermediate succinate is linked to the inflammatory activity of macrophages [127]. Extracellular succinate can activate succinate receptor SUCNR1 (or GPR91) to induce IL-1β production in macrophages during the inflammatory microenvironment [128]; yet conversely, macrophage-secreted succinate can also interact with neural stem cell SUCNR1 to promote PGE2 secretion for ameliorating chronic neuroinflammation [129]. Moreover, a recent study has demonstrated that cancer cells also secrete succinate to activate the succinate receptor and HIF-1α signaling axis in tumor-associated macrophages (TAMs) that can control M2 polarization and immunosuppression for tumorigenesis [130]. In addition to succinate, itaconate diverted away from cis-aconitate in the TCA cycle is mediated by immune-responsive gene 1 protein (IRG1), and functions similarly to malonate to disrupt SDH activity. This results in the accumulation of succinate and remodeling of immunoactivation in macrophages [101,131]. Furthermore, high levels of itaconate are present in TAMs and deficiency of IRG1 markedly reduces this scenario, impairing the mitochondrial respiratory activity of TAMs and thereby effectively stunting tumor progression in the peritoneal cavity [132]. Mechanistically, itaconate activates a nuclear factor erythroid 2-related factor 2 (Nrf2) via alkylation of Kelch ECH associating protein 1 (KEAP1) to limit inflammation and type I interferon for anti-inflammatory macrophage response [133].

As mentioned previously, α-KG is an important intermediate from the TCA cycle that is appreciated not only as a metabolite, but also a cofactor for several epigenetic-modifying enzymes involved in histone or DNA demethylation [134,135]. Glutaminolysis of glutamine can generate α-KG that is essential to sustain mitochondrial fitness and promote M2 activation [66]. Further, glutamine-derived α-KG has been shown to reprogram JMJD3-mediated demethylation of H3K27 at the promoters of genes specific to M2 macrophages [65]. Additionally, α-KG is also generated as a byproduct from serine biosynthesis in which 3-phosphohydroxypyruvate is catabolized into 3-phosphoserine by PSAT1. This suggests that the serine biosynthesis cascade may provide an alternative route of α-KG for downstream metabolism and/or support the activity of α-KG-dependent regulations. Although it has recently been indicated that serine metabolism is important to support T cell function [76,77] and IL-1β production in pro-inflammatory macrophages [136,137], the role of PSAT1 in immune cells is still hazy.

In addition to generating α-KG, glutamine is known to be a necessary substrate for generating UDP-GlcNAc via the hexosamine biosynthesis pathway to promote protein glycosylation during polarization [66,138]. M2 macrophages do not exclusively rely on glutamine uptake for their function, but they induce cell-intrinsic glutamine synthesis from glutamate via glutamine synthetase (GS). Inhibition of GS leads to induction of NO, IL-6, and IL-12 secretion by microglia during inflammation [139]. It was found that TAMs have induced GS expression, and attenuation of GS decreases the M2 phenotype and lessens tumor metastasis in animal models [116,140].

The resolution of macrophage inflammation is mediated by tryptophan metabolism through IDO and tryptophan 2, 3-dioxygenase (TDO). TAMs have been found to scavenge tryptophan and express high levels of IDO and TDO to decrease tryptophan availability in the tumor microenvironment [141], which in turn promotes immunosuppressive M2 immunity to suppress T cell function [64]. Importantly, in addition to starving T cells of tryptophan, IDO and TDO can metabolize tryptophan to kynurenine, the accumulation of which can lead to reduced effector T cell proliferation [142] and dendritic cell priming [143], but increase regulatory T cell numbers [142].

While each cell undergoes its specific metabolic processes, systemic metabolism is carried out across tissues and organs, and liver is one of the major venues to participate in carbohydrate (e.g., glycogenolysis, glycogenesis, fructose metabolism, galactose metabolism) and amino acid metabolism (e.g., glucose-alanine cycle) [39,144,145]. Kupffer cells (KCs) are tissue-specific resident macrophages and are known to regulate tissue homeostasis and metabolism in the liver [146]. KCs can express a range of polarization markers from pro-inflammatory M1 to anti-inflammatory M2, and this plasticity and heterogeneity is crucial for hepatic immune response and is tightly associated with various metabolic disorders including obesity [146]. In the lean liver, KCs tend to exhibit an M2-like phenotype, coordinating with other immune cells to aid in the function of tissue homeostasis and repair [147,148]. Conversely, greater fat accumulation in the liver will skew KCs into an M1 phenotype leading to an increased production of pro-inflammatory cytokines (e.g., TNF-α, IL-6, and IL-1β), chemokines (e.g., MCP-1), and prostaglandins (e.g., PGE2) which exacerbate the hepatic inflammatory response and perturb liver homeostasis [147,148,149]. Moreover, inflammatory activation of KCs has been associated with obesity-induced insulin resistance and fatty liver disease. As reported previously, depletion of hepatic macrophages can protect against insulin resistance, where TNF-α serves as an important mediator of this effect [150]. On the other hand, alternative activation of KCs has been considered to ameliorate obesity-induced insulin resistance in a PPARδ-dependent manner [151].

### 2.3. Dendritic Cells (DCs)

Like macrophages, DCs are present in nearly all tissues of body and serve as professional antigen presenting cells which help initiate antigen-specific adaptive immune responses. DCs exhibit heterogeneity in cell marker and distinct capabilities for engagement of effector T cells [152]. DCs express a range of pattern recognition receptors (PRRs) which can sense danger signals. Importantly, DCs can process peptide epitopes onto MHC class I or II molecule to stimulate T cells and effectively evoke adaptive immune response. It is increasingly clear that different DC subsets exhibit different metabolic dependencies to support their activation and function [153]. An increased reliance on mitochondrial activity and FAO for energy production has been associated with GM-CSF generated BMDCs or tolerogenic DCs, which were shown to reduce T cell engagement and activation [154,155]. However, upon TLR stimulation, activated DCs manifest enhanced glycolytic metabolism with increased glucose consumption and lactate production [156,157], and inhibition of glycolysis by 2-DG strongly blocks DC activation [154,158]. Akt and HIF-1α pathway are involved in the regulation of DC activation [11,57]. mTORC1 positively regulates Akt signaling in DC activation, and antagonization of mTORC1 selectively impairs cell activation and cytokine secretion in TLR-activated BMDCs and human DCs [159,160,161]. Activation of Akt is dependent on TANK-binding kinase 1 (TBK1) and inhibitor of NF-κB kinase subunit-ε (IKKε) which can directly phosphorylate and activate hexokinase II (HK2) to promote glycolysis for rapid DC activation in response to environmental danger cues [158]. It has been reported that TLR stimulation increases the expression of HIF-1α, promoting glycolysis and activation of DCs [156]. Additionally, HIF-1α but not mTORC1 can mediate the sirtuin 1 (SIRT1)-dependent signaling axis for the production of IL-12 and TGF-β1 in DCs which in turn mediate T cell differentiation [162]. Further, recent work has illustrated that glycolytic metabolism is essential for DC motility and CCR7-dependent migration to lymph nodes [163]. Intriguingly, however, this migration can be terminated by the intrinsic feedback regulation of long noncoding RNA (lnc)-Dpf3 to inhibit HIF-1α-mediated glycolysis in DCs [164]. In addition, a recent study reported that apart from glucose, DCs can also utilize cell-intrinsic glycogen stores to fuel basal glycolytic demands and support their immune effector function, particularly at early stages of activation and in glucose-restricted conditions [165].

Plasmacytoid DCs (pDCs) are a rare type of immune cells known to express a limited number of PRRs (i.e., TLR7 and 9) but at the same time, are the most efficient cells to produce type I interferon for antiviral and/or anti-cancer responses [166]. Mouse pDCs generated from FMS-like tyrosine kinase 3 ligand (FLT3L) stimulation display an increase of mitochondrial FAO and OXPHOS upon exposure to IFN-α. Importantly, elevated mitochondrial lipid catabolism is regulated by PPAR-α and administration of FAO inhibitor significantly disrupts IFN-α production of pDCs leading to higher viral loads in animals [167]. Respiratory viral infection was showed to increase the activity of glycolysis in human pDCs, and inhibition of which attenuates pDC antiviral responses [168]. In addition, it has been indicated that immunostimulatory function of intratumoral pDCs is affected by the high level of extracellular lactate promoting immunosuppression in the tumor microenvironment [169].

Similar to macrophages, catabolism of arginine and tryptophan are critical for DCs to balance inflammation and tolerance. This is especially apparent in the tumor microenvironment where DCs can be educated toward a protumoral tolerogenic phenotype, characterized by low costimulatory molecule expression, poor antigen presentation, and high expression of Arg1 and IDO [170,171]. pDCs has been reported to accumulate in tumor-draining lymph nodes that constitutively express IDO to mediate immunosuppression against T cell function [172]. It is known that tumor-derived PGE2 can impair the function of dendritic cells resulting in cancer immune evasion [173]. PGE2 increases the expression of Arg1 to promote tolerogenic phenotype in DCs to inhibit the proliferation of CD4+ T cells in the tumor microenvironment [174]. Further, IL-6 was also found to induce the expression of Arg1 leading to downregulation of MHC-II in DCs to dysregulate T cell immunity in tumors [175]. These Arg1-expressing tumor-infiltrating DCs also contribute to local arginine depletion and indirectly dampen T cell anti-tumor response [176].

### 2.4. Neutrophils

Neutrophils are the most abundant leukocytes in the circulation, are fully differentiated, and have a relatively short lifespan. As one of the first line in host defense, neutrophils circulate to damaged tissue and initiate an anti-pathogenic response including phagocytosis, extracellular ROS production, neutrophil extracellular trap (NET) production, and cytotoxic granule release [177,178]. However, the metabolic reprogramming in the regulation of neutrophil development and function is still not fully understood.

Neutrophils have been traditionally considered to selectively utilize glycolysis for energy metabolism due to having only a few mitochondria [179]. Extrinsic glucose is taken up into human neutrophils via GLUT1, which is expressed and upregulated in glucose-rich environments [180]. Patients with genetic deficiency in enzymes related to glucose metabolism suffer from neutrophil dysfunctions and neutropenia [20,27,180,181]. Neutrophils from glycogen storage disease (GSD) patients having glucose-6-phosphate transporter (G6PT) deficiency and manifest dysregulated function of energy homeostasis, ROS production, and chemotaxis, suggesting the importance of glucose metabolism in neutrophils [27]. Although mitochondrial bioenergetics is dispensable for neutrophils, mitochondrial proteins have been observed to regulate their motility. Deficiency of mitochondrial enzymes superoxide dismutase (SOD1), DNA polymerase (POLG), and IDH1 impaired neutrophil chemotaxis, suggesting the importance of mitochondria for neutrophil chemotaxis during inflammation [182,183,184].

Furthermore, it has been shown that neutrophil phagocytic function predominantly depends on glycolytic metabolism, as glycolytic inhibition, but not mitochondrial respiration, has a significant impact on phagocytizing neutrophils, suggesting a dispensable role of mitochondrial metabolism to neutrophil phagocytosis [185,186]. The production of NADPH by glucose-dependent PPP was shown to be essential for the cytosolic NOX-dependent ROS production for NET formation, which allow neutrophils to prevent the dissemination of pathogenic insults [187]. G6PD is a key enzyme that shifts glucose metabolism toward PPP, and patients with G6PD deficiency exhibit such as deficient bacterial killing and chronic granulomatous disease due to dysregulated metabolic activity of neutrophils [188,189]. It has been demonstrated that neutrophils can also utilize glutamine [190,191] and is similar to PPP which is important for the generation of NADPH and the expression of NOX [192]. Interestingly, however, utilization of extracellular glucose, but not glutamine, can protect human neutrophils from spontaneous and anti-Fas antibody-induced apoptosis [193].

Autophagy has a crucial role in providing metabolites (e.g., amino acids, free fatty acids) as substrates for biomolecule synthesis and energy generation in cells [194]. Autophagy-deficient neutrophils display decreased mitochondrial fitness and pronounced lipid accumulation, which lead to differentiation defects and metabolic crisis [195]; however, the mechanism of how metabolic networks shape neutrophil development and homeostasis remains elusive.

### 2.5. Myeloid-Derived Suppressor Cells (MDSCs)

MDSCs constitute a heterogeneous population of two major subsets, monocytic (M)-MDSCs and granulocytic (G)-MDSCs, which share an immature myeloid phenotype and the ability to suppress adaptive immune response in a number of inflammatory environments, including cancer [196], infections [197], and autoimmune diseases [198].

A metabolic hallmark of MDSCs is the increase of cellular amino acid metabolism. MDSCs control immune tolerance using different mechanisms, one of which is by depleting metabolites from the extracellular space, such as arginine, tryptophan, and cysteine, and preventing their uptake by T cells [199]. Metabolic conversion of arginine via either Arg1 or iNOS is the key mechanism strongly associated with MDSC immunosuppressive property. The increased activity of Arg1 in MDSCs results in deprivation of arginine from the environment and inhibits T cell proliferation; whereas, iNOS-mediated NO generation affects T cell effector function and induces cell apoptosis [200]. MDSCs were shown to engage in cystine uptake which reduces the extracellular cystine pool and subsequently limits the availability of cysteine required for T cell activation [201]. Additionally, it has been demonstrated that MDSCs express IDO that degrades tryptophan to kynurenines, promoting the expression of aryl hydrocarbon receptor (AHR) for Treg expansion [202] and impeding DC immunostimulatory activity [203].

MDSCs seem to exhibit a higher level of glycolysis than their mature myeloid counterparts in the tumor microenvironment, and this higher glycolytic activity is able to dampen excess ROS production contributing to the survival of MDSCs in the tumor [204]. HIF-1α upregulation has also been shown to significantly govern the suppressive activity of MDSCs in the tumor microenvironment [205]. Moreover, tumor-derived lactate has been suggested to directly impair the cytotoxic function of NK and T cells and can control MDSC development and increase cell numbers [206,207,208]. Tumor-associated MDSCs were shown to adapt lipid metabolism as fuel via the upregulation of lipid uptake and mitochondrial fatty acid oxidation to enhance their inhibitory cytokine production in cancer [209,210]. Yet, it is still not fully understood what specific regulatory factors facilitate MDSC metabolic adaption shift from glycolysis to mitochondrial FAO for controlling their immunoinhibitory role in the tumor milieu.

## 3. Concluding Remarks

Myriad findings from the past two decades have solidified the relationship between metabolism and immunity, and it continues to become clear that these two systems are intimately connected. Cells of the innate immune system in particular have evolved conserved metabolic pathways, and utilize these pathways similarly depending on signals from the surrounding environment. Increased glycolysis seems to be induced in the context of inflammation, where the quick energy turnover and focus on pathways involving ROS and antioxidants favor phenotypes associated with phagocytosis, pathogen killing, and antigen presentation. Conversely, enhanced mitochondrial respiration and oxidative TCA cycle reaction have been associated with the resolution of inflammation, wound healing/tissue homeostasis, and poorer antigen presentation. We have also learned that key modulators of these pathways, glucose and amino acids, drive the expression of these phenotypes. Yet, despite this paradigm, we have also learned that these networks can become dysregulated. Loss of carbohydrates and amino acids result in activation failure in a number of the cell types discussed, and inhibition of key enzymes along these pathways can prevent or significantly impair effector function.

While great strides have been made toward understanding how metabolism interfaces with immunity, there is still much that is unclear. We have only begun to appreciate how cell metabolites including amino acids contribute to epigenetic regulation in innate immune cells, and further study is needed to uncover how this signaling axis promotes gene expression of key cytokines associated with activation state. Many of these metabolic enzymes and products have become of interest as therapeutic targets for diabetes, obesity, and cancer. Employing targeted approaches to intervene metabolic programs or rewire dysfunctional pathways could become a novel means by which to enhance current immunotherapies and improve therapeutic outcomes.

## Figures and Tables

**Table 1:** Summary of immunological and metabolic features in M1 and M2 macrophages. AMPK, AMP-activated protein kinase; ETC, electron transport chain; FAO, fatty acid oxidation; FAS, fatty acid synthase; HIF-1α, hypoxia-inducible factor 1α; IL-1Ra, interleukin 1 receptor antagonist; iNOS, inducible nitric oxide synthase; mTORC1, mechanistic target of rapamycin complex 1; mTORC2, mechanistic target of rapamycin complex 2; OXPHOS, oxidative phosphorylation; PGC1β, peroxisome proliferative activated receptor gamma coactivator 1 beta; PPAR-γ, peroxisome proliferator-activated receptor gamma; PPP, pentose phosphate pathway; TCA cycle, tricarboxylic acid cycle.

Cell Type	Inducers	Activation Markers	Cytokine/Chemokine Production	Metabolic Pathways	Cellular Signaling Pathways	Functions
M1	LPS/IFN-γ	CD80, CD86, MHC-II, CCR7, iNOS	IL-1β, IL-6, IL-12, IL-15, IL-23, TNF-α, CCL3, CCL5, CXCL10	Glycolysis, PPP, Broken TCA cycle, FAS	NF-kB, PI3K/Akt, mTORC1, HIF-1α, STAT1, IRF5	Killing intracellular pathogens; Anti-tumor immunity
M2	IL-4/IL-13	CD206, CD301, PD-L2, RELMα, CD163, Arg1	IL-10, TGF-β, IL-1Ra, CCL17, CCL22, CCL24	Glycolysis, OXPHOS/ETC, FAO, Glutaminolysis	PI3K/Akt, AMPK, PGC1β PPAR-γ mTORC1, mTORC2, STAT6, IRF4	Tissue repair; Anti-helminth immunity; Pro-tumor activity
